# Changing Patterns of Disease and Mortality at the Children’s Hospital, Accra: Are Infections Rising?

**DOI:** 10.1371/journal.pone.0150387

**Published:** 2016-04-05

**Authors:** Edem M. A. Tette, Margaret Neizer, Maame Yaa Nyarko, Eric K. Sifah, Edmund T. Nartey, Eric S. Donkor

**Affiliations:** 1 Department of Community Health, School of Public Health, University of Ghana, Legon, Ghana; 2 Princess Marie Louis Children’s Hospital, Accra, Ghana; 3 Centre for Tropical Clinical Pharmacology and Therapeutics, School of Medicine and Dentistry, University of Ghana, Legon, Ghana; 4 Department of Epidemiology and Disease Control, School of Public Health, University of Ghana, Legon, Ghana; 5 Department of Medical Microbiology, College of Health Sciences, University of Ghana, Legon, Ghana; Instituto de Higiene e Medicina Tropical, PORTUGAL

## Abstract

**Background:**

The Millennium Development Goals (MDGs) have led to reductions in child mortality world-wide. This has, invariably, led to the changes in the epidemiology of diseases associated with child mortality. Although facility based data do not capture all deaths, they provide an opportunity to confirm diagnoses and insight into these changes which are relevant for further disease control.

**Objective:**

To identify changes in the disease pattern of children who died at the Princess Marie Louise Children’s Hospital (PML) in Ghana from 2003–2013.

**Methods:**

A cross sectional review of mortality data was carried out at PML. The age, sex, duration of admission and diagnosis of consecutive patients who died at the hospital between 2003 and 2013 were reviewed. This information was entered into an Access database and analysed using Stata 11.0 software.

**Results:**

Altogether, 1314 deaths (3.6%) occurred out of a total of 37,012 admissions. The majority of the deaths, 1187 (90.3%), occurred in children under the age of 5 years. While deaths caused by malaria, malnutrition, HIV infection and diarrhoea decreased, deaths caused by pneumonia were rising. Suspected septicaemia and meningitis showed a fluctuating trend with only a modest decrease between 2012 and 2013. The ten leading causes of mortality among under-fives were malnutrition, 363 (30.6%); septicaemia, 301 (25.4%); pneumonia, 218 (18.4%); HIV infection, 183 (15.4%); malaria, 155 (13.1%); anaemia, 135 (11.4%); gastroenteritis/dehydration, 110 (9.3%); meningitis, 58 (4.9%); tuberculosis, 34 (2.9%) and hypoglycaemia, 27 (2.3%). For children aged 5–9 years, the leading causes of mortality were malaria, 42 (42.9%); HIV infection, 27 (27.6%); anaemia, 14 (14.3%); septicaemia, 12 (12.2%); meningitis, 10 (10.2%); malnutrition, 9 (9.2%); tuberculosis, 5 (5.1%); pneumonia, 4 (4.1%); encephalopathy, 3 (3.1%); typhoid fever, 3 (3.1%) and lymphoma, 3 (3.1%). In the adolescent age group, malaria, 8 (27.6%); anaemia, 6 (20.7%); HIV infection, 5 (17.2%); sickle cell disease, 3 (10.3%) and meningitis, 3 (10.3%) were most common.

**Conclusion:**

There has been a decline in the under-five mortality at PML over the years; however, deaths caused by pneumonia appear to be rising. This highlights the need for better diagnostic services, wider HIV screening and clinical audits to improve outcomes in order to achieve further reductions in child mortality and maintain the gains.

## Introduction

The Millennium Development Goals have succeeded in reducing child mortality by almost half globally [1]. This has led to changes in the patterns of diseases which cause mortality in under-fives [2]. According to the Ghana Demographic and Health Survey, 2008, the major causes of child mortality in Ghana were neonatal disorders (27%), malaria (25%), pneumonia (20%), diarrhoeal diseases (17%), HIV/AIDS (8%) and measles (3%) [3]. However, by 2012, the major causes of child mortality in Ghana had changed to neonatal disorders (40%), malaria (19%), pneumonia (11%), diarrhoeal diseases (7%), HIV/AIDS (1%), injuries 4%, measles (1%) and others (17%) [1]. It is not clear how the pattern of disease and mortality has changed in school-aged children and adolescents.

Child mortality from Korle Bu Teaching Hospital, the country’s premier hospital, using data from the 1980s, showed that overall, neonatal deaths accounted for almost half (49.2%) of the total deaths with prematurity being the leading cause and accounting for 21% of the deaths [4]. This was followed by protein energy malnutrition (6.9%) and gastroenteritis (6.5%). Cerebral malaria was the seventh most common cause of death accounting for 5.4%. Unfortunately, although hospital-based studies provide a means of capturing these changes, published studies on current data from Ghana and other developing countries are largely unavailable.

We examined the causes of deaths in children admitted to the Princess Marie Louise Children’s Hospital over an 11 year period from 2003–2013 to determine changing trends and their implications for further disease control.

## Materials and Methods

### Ethical clearance

We obtained ethical clearance from the Ghana Health Service Ethical Review Committee (ID NO: GHS-ERC: 05/07/12). We could not obtain consent from the patient’s caregivers. However, patient information was anonymized and de-identified prior to the analysis.

### Study area

Princess Marie Louise Children’s Hospital (PML) was the site for the study. It is a 74 bed hospital and the second largest paediatric facility in Accra, located at the commercial centre of the capital city. It has the largest Nutritional Rehabilitation Unit in the country. Princess Marie Louise Children’s Hospital provides both primary and secondary care for paediatric patients under the age of 18 years, in accordance with the definition of a child in the Children’s ACT Ghana, 1998 [5]. Thus, parents can bring their children to the hospital with or without a referral at any time. Paediatric referrals are received from health centres, private clinics, government polyclinics and hospitals located in and around Accra including Korle Bu Teaching Hospital. The latter is the largest tertiary referral unit in Ghana and it is 15–30 minutes’ drive in normal traffic from PML. A small proportion of patients are referred to PML from outside Accra.

Mortality meetings are held regularly at PML to audit the deaths that occur at the hospital. Mortality data collected at the time are recorded and stored at the Records Department. X-ray services are available from 8am to 2pm daily. Full blood counts, blood film for malaria parasites, sickling test, G6PD test, Hb Electrophoresis, urinalysis and stool examination are among the tests done by the laboratory on site. Rapid diagnostic tests for malaria parasites became regularly available in 2013. There are no facilities for bacterial cultures and antibiotic susceptibility testing. Requests for these are normally sent to the Korle Bu Teaching Hospital or private laboratories in Accra.

### Study design

This was a retrospective review of consecutive deaths occurring in children aged 0–17 years admitted to the Princess Marie Louise Hospital from 1^st^January, 2003 to 31^st^December, 2013. This study was part of a broader child mortality study, which examined general trends in mortality, place of residence and mortality, and compared under-five deaths and discharges in 2011. These are being presented elsewhere. We report here only the top ten causes of death in these children.

### Study population

We included all patients under the age of 18 who died at the hospital from 1^st^ January, 2003 to 31^st^ December, 2013. They were further categorized into <5 years, 5–9 years and 10–17 years. The total admissions for each year were also obtained.

### Data collection methods and instruments

Children who die at the hospital are first discussed at weekly and monthly mortality meetings. A paediatrician then fills in the death certificate and a data sheet for the data management unit of the hospital which include the clinical diagnosis at the time of death and some selected information. This is based on the cause of death agreed at the mortality meetings and it is usually supported by preliminary laboratory tests that have been done or the results of any other investigations to confirm the diagnosis. This process became operational at PML in 2009. Thus, the causes of death presented from then are based on consensus from the mortality meetings.

Prior to this, the mortality meetings were held monthly and involved only medical staff. Collection of mortality data was driven by data management personnel of the Records Department, with less co-ordination between them and the paediatricians. The list of children who died was compiled by these data management personnel using information obtained from admissions, death/discharge books and death certificates. This list together with the case notes was then given to a doctor to confirm the diagnosis.

Since 2008, morbidity and mortality data has been entered into the District Health Information Management System (DHIMS 1) and DHIMS 2 from 2012 [6] using manually complied data master sheets. This is a nationwide database for recording in-patient and out-patient morbidity and mortality information using a standard format including diagnostic categories for the top 10 diseases. Selected information is collected on each in-patient including causes of admission and death with some International Statistical Classification of Diseases and Related Health Problems-10th Revision (ICD 10) coded diagnosis from the DHIS 2 Tracker. The data presented here includes, mostly data from the manually collated data master sheets held by the data management unit of PML and some print-outs from the DHIMS.

Standard case definitions from the World Health organisation (WHO) are routinely used for the diagnosis of diarrhoea, pneumonia, malaria, anaemia and malnutrition [7,8]. In addition to this, case definitions for cholera and measles are provided by the Ghana Health Service when there is an outbreak and are based on WHO Integrated Disease Surveillance and Response Guidelines [9]. The rest comes from the physician’s clinical practice.

We obtained the records of all deaths occurring from 2003 to 2013 from the Records Department of the hospital and entered them into a computerised record form. This was done by trained data management personnel who were recruited by the investigators to enter information on the printouts and data sheets. We did not go back to review the case notes of the patients. The information collected included data on the age, sex, cause of death, duration of admission, status of registration with the National Health Insurance Scheme and place of residence. The total admissions for each year were obtained to enable the deaths to be expressed as a percentage of the admissions.

All causes of death were recorded. Some patients had multiple diagnoses and each of these diagnoses was reported separately. This was done in order to provide a better impression of the burden of disease as some of these patients were treated for different diseases and billed for their treatments. In addition, since this was a retrospective study, we felt that this was the best way to capture the disease burden as mentioned by other researchers studying child mortality from the developing world [10].

### Data analysis

The data was summarized using Microsoft Access (Microsoft Corporation, Edmond, Washington) and analyzed using Stata SE 11.0 (Stata Corporation, College Station, Texas). We determined the frequencies and proportion of the top ten diseases and presented them in tables according to the following age groups: 0–4 years (under-fives), 5–9 years and 10–17 years. Mortality ratios were calculated using data on the number of deaths per 1000 age-specific admissions. Each disease category was also presented as a percentage of total deaths.

## Results

Out of a total of 37,012 admissions within the period of this study, 1,314 deaths occurred, giving a death rate of 3.6% of total admissions (range 2.6% to 6.3%). This does not include data on child deaths that occurred in June 2003, February 2007 and March 2007 as they could not be found. The ages ranged between 1 day and 15 years and 52.2% (686) of the children who died were males. Under-fives formed 90.3%, (1187) of the children who died, while children aged five years and above formed 9.7% (127). Ninety-eight deaths occurred within the first month of life and they formed 7.5% of the under-five mortality.

Altogether, 676 (51.4%) of the children had one cause of death, while 435 (33.1%) had two causes of death and 203 (15.5%) children had three or more causes of death.

Table 1 shows the proportionate distribution (per 1000 admissions) of ten leading causes of death in under-fives whereas Table 2 shows the percentage distribution of ten leading causes of death in under-fives. The proportion of children dying from malnutrition reduced from 32.4 deaths per 1000 total admission in 2003 to 7.2 deaths per 1000 total admission in 2008 and 6.1 per 1000 total admission in 2013 (Table 1). Similar trends were observed for malaria, diarrhoea and HIV infection. The proportion of children under-five years who died from pneumonia increased during the review period. However, the proportion of under-fives who died from septicaemia and meningitis fluctuated during the review period. The ten leading causes of mortality in children under the age of 5 years were malnutrition (30.6%), septicaemia (25.4%), pneumonia (18.4%), HIV Infection (15.4%), malaria (13.1%), anaemia (11.4%), gastroenteritis/dehydration (9.3%), meningitis (4.9%), tuberculosis (2.9%) and hypoglycaemia (2.3%) (Table 2).

**Table 1:** Proportionate distribution (per 1000 admissions) of ten leading causes of death in patients under 5 years attending PML hospital in Accra, Ghana, 2003–2013.

	Total	2003^b^	2004	2005	2006	2007^c^	2008	2009	2010	2011	2012	2013
Cause of death^a^	N = 32784	N = 1450	N = 1768	N = 1749	N = 1951	N = 2272	N = 2796	N = 3386	N = 3936	N = 4677	N = 4621	N = 4178
	n, p	n, p	n, p	n, p	n, p	n, p	n, p	n, p	n, p	n, p	n, p	n, p
Malnutrition	363 (11.1)	47 (32.4)	48 (27.1)	31 (17.7)	20 (10.3)	16 (7.0)	20 (7.2)	32 (9.5)	40 (10.2)	40 (8.6)	40 (8.7)	29 (6.9)
Septicaemia	301 (9.2)	2 (1.4)	19 (10.7)	9 (5.1)	4 (2.1)	9 (4.0)	25 (8.9)	39 (11.5)	53 (13.5)	50 (10.7)	55 (11.9)	36 (8.6)
Pneumonia	218 (6.6)	5 (3.4)	17 (9.6)	5 (2.9)	7 (3.6)	14 (6.2)	13 (4.6)	23 (6.8)	37 (9.4)	37 (7.9)	26 (5.6)	34 (8.1)
HIV (Positive/Exposed)	183 (5.6)	9 (6.2)	14 (7.9)	18 (10.3)	18 (9.2)	30 (13.2)	24 (8.6)	12 (3.5)	20 (5.1)	15 (3.2)	9 (1.9)	14 (3.4)
Malaria	155 (4.7)	18 (12.4)	5 (2.8)	3 (1.7)	2 (1.0)	11 (4.8)	22 (7.9)	27 (8.0)	26 (6.6)	15 (3.2)	17 (3.7)	9 (2.2)
Anaemia	135 (4.1)	10 (6.9)	6 (3.4)	4 (2.3)	1 (0.5)	9 (4.0)	15 (5.4)	15 (4.4)	25 (6.4)	16 (3.4)	20 (4.3)	14 (3.4)
Gastroenteritis with/without dehydration	110 (3.4)	5 (3.4)	10 (5.7)	6 (3.4)	-	6 (2.6)	8 (2.9)	13 (3.8)	12 (3.0)	18 (3.8)	20 (4.3)	12 (2.9)
Meningitis	58 (1.8)	1 (0.7)	4 (2.3)	1 (0.6)	-	1 (0.4)	5 (1.8)	4 (1.2)	14 (3.6)	12 (2.6)	10 (2.2)	6 (1.4)
Tuberculosis	34 (1.0)	-	3 (1.7)	3 (1.7)	1 (0.5)	-	5 (1.8)	3 (0.9)	4 (1.0)	8 (1.7)	5 (1.1)	2 (0.5)
Hypoglycaemia	27 (0.8)	-	-	-	-	-	-	-	6 (1.5)	6 (1.3)	9 (1.9)	6 (1.4)

^a^ Cause of death may be multiple in some cases

^b^ Data on patients who died in June 2003 missing

^c^ Data on patients who died in February and March 2007 missing; N = Total number of admissions in each year; n = number of deaths; p = proportionate death per 1000 year-specific admissions

**Table 2:** Percentage distribution of ten leading causes of death in patients under 5 years attending PML hospital in Accra, Ghana, 2003–2013.

	Total	2003^b^	2004	2005	2006	2007^c^	2008	2009	2010	2011	2012	2013
Cause of death^a^	N = 1187	N = 95	N = 112	N = 72	N = 55	N = 94	N = 119	N = 119	N = 137	N = 130	N = 141	N = 113
	n, %	n, %	n, %	n, %	n, %	n, %	n, %	n, %	n, %	n, %	n, %	n, %
Malnutrition	363 (30.6)	47 (49.5)	48 (42.9)	31 (43.1)	20 (36.4)	16 (17.0)	20 (16.8)	32 (26.9)	40 (29.2)	40 (30.8)	40 (28.4)	29 (25.7)
Septicaemia	301 (25.4)	2 (2.1)	19 (17.0)	9 (12.5)	4 (7.3)	9 (9.6)	25 (21.0)	39 (32.8)	53 (38.7)	50 (38.5)	55 (39.0)	36 (31.9)
Pneumonia	218 (18.4)	5 (5.3)	17 (15.2)	5 (6.9)	7 (12.7)	14 (14.9)	13 (10.9)	23 (19.3)	37 (27.0)	37 (28.5)	26 (18.4)	34 (30.1)
HIV (Positive/Exposed)	183 (15.4)	9 (9.5)	14 (12.5)	18 (25.0)	18 (32.7)	30 (31.9)	24 (20.2)	12 (10.1)	20 (14.6)	15 (11.5)	9 (6.4)	14 (12.4)
Malaria	155 (13.1)	18 (18.9)	5 (4.5)	3 (4.2)	2 (3.6)	11 (11.7)	22 (18.5)	27 (22.7)	26 (19.0)	15 (11.5)	17 (12.1)	9 (8.0)
Anaemia	135 (11.4)	10 (10.5)	6 (5.4)	4 (5.6)	1 (1.8)	9 (9.6)	15 (12.6)	15 (12.6)	25 (18.2)	16 (123.3)	20 (14.2)	14 (12.4)
Gastroenteritis with/without dehydration	110 (9.3)	5 (5.3)	10 (8.9)	6 (8.3)	-	6 (6.4)	8 (6.7)	13 (10.9)	12 (8.8)	18 (13.8)	20 (14.2)	12 (10.6)
Meningitis	58 (4.9)	1 (1.1)	4 (3.6)	1 (1.4)	-	1 (1.1)	5 (4.2)	4 (3.4)	14 (10.2)	12 (9.2)	10 (7.1)	6 (5.3)
Tuberculosis	34 (2.9)	-	3 (2.7)	3 (4.2)	1 (1.8)	-	5 (4.2)	3 (2.5)	4 (2.9)	8 (6.2)	5 (3.5)	2 (1.8)
Hypoglycaemia	27 (2.3)	-	-	-	-	-	-	-	6 (4.4)	6 (4.6)	9 (6.4)	6 (5.3)

^a^ Cause of death may be multiple in some cases, hence %s may add up to >100

^b^ Data on patients who died in June 2003 missing

^c^ Data on patients who died in February and March 2007 missing; N = Total number of deaths in each year; n = number of deaths

Table 3 shows the proportionate distribution (per 1000 admissions) of ten leading causes of death in patients aged 5–9 years whereas Table 4 shows the percentage distribution of ten leading causes of death in patients aged 5–9 years. Table 3 indicates a drop in malaria mortality from 19.4 deaths per 1000 total admissions in 2003 to 4.7 deaths per 1000 total admissions in 2013. In 2013 only confirmed malaria cases were reported whereas in the previous years, data on suspected and confirmed cases were not separated. The top ten causes of death in children aged 5–9 years were malaria 42 (42.9%), HIV 27 (27.6%), anaemia 14 (14.3%), septicaemia 12 (12.2%), meningitis 10 (10.2%), malnutrition 9 (9.2%), tuberculosis 5 (5.1%), pneumonia 4 (4.1%), encephalopathy 3 (3.1%), typhoid fever 3 (3.1%) and three (3) cases of lymphoma (3.1%) (Table 4).

**Table 3:** Proportionate distribution (per 1000 admissions) of ten leading causes of death in patients aged 5–9 years attending PML hospital in Accra, Ghana, 2003–2013.

	Total	2003^b^	2004	2005	2006	2007^c^	2008	2009	2010	2011	2012	2013
Cause of death^a^	N = 3337	N = 103	N = 164	N = 149	N = 168	N = 242	N = 357	N = 336	N = 468	N = 486	N = 441	N = 423
	n, p	n, p	n, p	n, p	n, p	n, p	n, p	n, p	n, p	n, p	n, p	n, p
Malaria	42 (12.6)	2 (19.4)	-	-	2 (11.9)	3 (12.4)	9 (25.2)	5 (14.9)	8 (17.1)	4 (8.2)	7 (15.9)	2 (4.7)
HIV (Positive/Exposed)	27 (8.1)	-	2 (12.2)	3 (20.1)	-	1 (4.1)	4 (11.2)	5 (14.9)	5 (10.7)	1 (2.1)	3 (6.8)	3 (7.1)
Anaemia	14 (4.2)	-	-	-	1 (6.0)	2 (8.3)	2 (5.6)	3 (8.9)	2 (4.3)	1 (2.1)	2 (4.5)	1 (2.4)
Septicaemia	12 (3.6)	-	-	-	-	-	1 (2.8)	1 (3.0)	3 (6.4)	2 (4.1)	4 (9.1)	1 (2.4)
Meningitis	10 (3.0)	-	1 (6.1)	-	-	-	1 (2.8)	1 (3.0)	6 (12.8)	1 (2.1)	-	-
Malnutrition	9 (2.7)	1 (9.7)	1 (6.1)	1 (6.7)	-	-	1 (2.8)	1 (3.0)	1 (2.1)	-	-	3 (7.1)
Tuberculosis	5 (1.5)	-	1 (6.1)	-	-	-	-	1 (3.0)	2 (4.3)	-	-	1 (2.4)
Pneumonia	4 (1.2)	-	-	-	1 (6.0)	-	-	1 (3.0)	-	1 (2.1)	1 (2.3)	-
Encephalopathy	3 (0.9)	-	-	-	-	-	-	1 (3.0)	-	-	2 (4.5)	-
Typhoid fever	3 (0.9)	-	-	-	-	1 (4.1)	-	1 (3.0)	-	1 (2.1)	-	-
Lymphoma	3 (0.9)	-	1 (6.1)	-	1 (6.0)	-	1 (2.8)	-	-	-	-	-

^a^ Cause of death may be multiple in some cases

^b^ Data on patients who died in June 2003 missing

^c^ Data on patients who died in February and March 2007 missing; N = Total number of admissions in each year; n = number of deaths; p = proportionate death per 1000 year-specific admissions

**Table 4:** Percentage distribution of ten leading causes of death in patients aged 5–9 years attending PML hospital in Accra, Ghana, 2003–2013.

	Total	2003^b^	2004	2005	2006	2007^c^	2008	2009	2010	2011	2012	2013
Cause of death^a^	N = 98	N = 3	N = 7	N = 4	N = 5	N = 6	N = 16	N = 13	N = 14	N = 6	N = 14	N = 10
	n, %	n, %	n, %	n, %	n, %	n, %	n, %	n, %	n, %	n, %	n, %	n, %
Malaria	42 (42.9)	2 (66.7)	-	-	2 (40.0)	3 (50.0)	9 (56.3)	5 (38.5)	8 (57.1)	4 (66.7)	7 (50.0)	2 (20.0)
HIV (Positive/Exposed)	27 (27.6)	-	2 (28.6)	3 (75.0)	-	1 (16.7)	4 (25.0)	5 (38.5)	5 (35.7)	1 (16.7)	3 (21.4)	3 (30.0)
Anaemia	14 (14.3)	-	-	-	1 (20.0)	2 (33.3)	2 (12.5)	3 (23.1)	2 (14.3)	1 (16.7)	2 (14.3)	1 (10.0)
Septicaemia	12 (12.2)	-	-	-	-	-	1 (6.3)	1 (7.7)	3 (21.4)	2 (33.3)	4 (28.6)	1 (10.0)
Meningitis	10 (10.2)	-	1 (14.3)	-	-	-	1 (6.3)	1 (7.7)	6 (42.9)	1 (16.7)	-	-
Malnutrition	9 (9.2)	1 (33.3)	1 (14.3)	1 (25.0)	-	-	1 (6.3)	1 (7.7)	1 (7.1)	-	-	3 (30.0)
Tuberculosis	5 (5.1)	-	1 (14.3)	-	-	-	-	1 (7.7)	2 (14.3)	-	-	1 (10.0)
Pneumonia	4 (4.1)	-	-	-	1 (20.0)	-	-	1 (7.7)	-	1 (16.7)	1 (7.1)	-
Encephalopathy	3 (3.1)	-	-	-	-	-	-	1 (7.7)	-	-	2 (14.3)	-
Typhoid fever	3 (3.1)	-	-	-	-	1 (16.7)	-	1 (7.7)	-	1 (16.7)	-	-
Lymphoma	3 (3.1)	-	1 (14.3)	-	1 (20.0)	-	1 (6.3)	-	-	-	-	-

^a^ Cause of death may be multiple in some cases, hence %s may add up to >100

^b^ Data on patients who died in June 2003 missing

^c^ Data on patients who died in February and March 2007 missing; N = Total number of deaths in each year; n = number of deaths

Table 5 shows the proportionate distribution (per 1000 admissions) of all the causes of death in patients aged 10–17 years whereas Table 6 shows the percentage distribution of all the causes of death in patients aged 10–17 years. There were no deaths recorded in this age category in 2013. Malaria was the leading cause of death accounting for 9 deaths per 1000 admissions (Table 5) which is 27.6% of all deaths recorded for the 10–17 year olds during the study period (Table 6). The other causes of death in the adolescent age group were anaemia, 6 (20.7%); HIV 5 (17.2%), sickle cell disease, 3 (10.3%) and meningitis, 3 (10.3%) (Table 6). There were two cases of malnutrition (6.9%) and two patients with unspecified diagnosis (6.9%) (Table 6). For the age group 10–17 years, we included all the causes of death because the number of deaths was small and not just the top ten leading causes of death.

**Table 5:** Proportionate distribution (per 1000 admissions) of causes of death in patients aged 10–17 years attending PML hospital in Accra, Ghana, 2003–2013.

	Total	2003^b^	2004	2005	2006	2007^c^	2008	2009	2010	2011	2012	2013
Cause of death^a^	N = 891	N = 31	N = 38	N = 42	N = 38	N = 75	N = 84	N = 83	N = 115	N = 120	N = 139	N = 126
	n, p	n, p	n, p	n, p	n, p	n, p	n, p	n, p	n, p	n, p	n, p	n, p
Malaria	8 (9.0)	-	-	-	-	-	3 (35.7)	2 (24.1)	1 (8.7)	1 (8.3)	1 (7.2)	-
Anaemia	6 (6.7)	-	-	-	1 (26.3)	-	-	2 (24.1)	2 (17.4)	-	1 (7.2)	-
HIV (Positive/Exposed)	5 (5.6)	-	-	1 (23.8)	-	2 (26.7)	-	1 (12.0)	-	1 (8.3)	-	-
Sickle cell disease	3 (3.4)	-	-	-	-	-	1 (11.9)	2 (24.1)	-	-	-	-
Meningitis	3 (3.4)	1 (32.3)	-	-	-	-	1 (11.9)	-	-	1 (8.3)	-	-
Malnutrition	2 (2.2)	-	-	-	-	-	-	-	1 (8.7)	-	1 (7.2)	-
Not specified	2 (2.2)	-	-	-	-	1 (13.3)	1 (11.9)	-	-	-	-	-
Septicaemia	2 (2.2)	-	-	-	-	-	1 (11.9)	-	-	1 (8.3)	-	-
Acute respiratory infection	2 (2.2)	-	-	-	-	-	-	1 (12.0)	-	-	1 (7.2)	-
Chest tumour	2 (2.2)	-	-	-	1 (26.3)	-	-	-	-	-	1 (7.2)	-
Pleural effusion	1 (1.1)	-	-	-	-	-	-	-	1 (8.7)	-	-	-
Gastroenteritis with/without dehydration	1 (1.1)	-	-	-	-	-	-	-	-	1 (8.3)	-	-
Acute chest syndrome	1 (1.1)	-	-	-	-	-	-	-	-	-	1 (7.2)	-
Intraventricular haemorrhage	1 (1.1)	-	-	-	-	-	-	1 (12.0)	-	-	-	-
Leukaemia	1 (1.1)	-	-	-	-	-	1 (11.9)	-	-	-	-	-
Pneumonia	1 (1.1)	-	-	-	-	-	-	-	1 (8.7)	-	-	-
Renal failure	1 (1.1)	-	-	-	-	1 (13.3)	-	-	-	-	-	-
Intracranial space occupying lesion	1 (1.1)	-	-	-	-	-	-	-	-	1 (8.3)	-	-
Tuberculosis	1 (1.1)	-	-	-	-	-	-	-	1 (8.7)	-	-	-

^a^ Cause of death may be multiple in some cases

^b^ Data on patients who died in June 2003 missing

^c^ Data on patients who died in February and March 2007 missing; N = Total number of admissions in each year; n = number of deaths; p = proportionate death per 1000 year-specific admissions

**Table 6:** Percentage distribution of causes of death in patients aged 10–17 years attending PML hospital in Accra, Ghana, 2003–2013.

	Total	2003^b^	2004	2005	2006	2007^c^	2008	2009	2010	2011	2012	2013
Cause of death^a^	N = 29	N = 1	N = 0	N = 1	N = 2	N = 3	N = 7	N = 6	N = 3	N = 3	N = 3	N = 0
	n, %	n, %	n, %	n, %	n, %	n, %	n, %	n, %	n, %	n, %	n, %	n, %
Malaria	8 (27.6)	-	-	-	-	-	3 (42.9)	2 (33.3)	1 (33.3)	1 (33.3)	1 (33.3)	-
Anaemia	6 (20.7)	-	-	-	1 (50.0)	-	-	2 (33.3)	2 (66.7)	-	1 (33.3)	-
HIV (Positive/Exposed)	5 (17.2)	-	-	1 (100)	-	2 (66.7)	-	1 (16.7)	-	1 (33.3)	-	-
Sickle cell disease	3 (10.3)	-	-	-	-	-	1 (14.3)	2 (33.3)	-	-	-	-
Meningitis	3 (10.3)	1 (100)	-	-	-	-	1 (14.3)	-	-	1 (33.3)	-	-
Malnutrition	2 (6.9)	-	-	-	-	-	-	-	1 (33.3)	-	1 (33.3)	-
Not specified	2 (6.9)	-	-	-	-	1 (33.3)	1 (14.3)	-	-	-	-	-
Septicaemia	2 (6.9)	-	-	-	-	-	1 (14.3)	-	-	1 (33.3)	-	-
Acute respiratory infection	2 (6.9)	-	-	-	-	-	-	1 (16.7)	-	-	1 (33.3)	-
Chest tumour	2 (6.9)	-	-	-	1 (50.0)	-	-	-	-	-	1 (33.3)	-
Pleural effusion	1 (3.4)	-	-	-	-	-	-	-	1 (33.3)	-	-	-
Gastroenteritis with/without dehydration	1 (3.4)	-	-	-	-	-	-	-	-	1 (33.3)	-	-
Acute chest syndrome	1 (3.4)	-	-	-	-	-	-	-	-	-	1 (33.3)	-
Intraventricular haemorrhage	1 (3.4)	-	-	-	-	-	-	1 (16.7)	-	-	-	-
Leukaemia	1 (3.4)	-	-	-	-	-	1 (14.3)	-	-	-	-	-
Pneumonia	1 (3.4)	-	-	-	-	-	-	-	1 (33.3)	-	-	-
Renal failure	1 (3.4)	-	-	-	-	1 (13.3)	-	-	-	-	-	-
Intracranial space occupying lesion	1 (3.4)	-	-	-	-	-	-	-	-	1 (33.3)	-	-
Tuberculosis	1 (3.4)	-	-	-	-	-	-	-	1 (33.3)	-	-	-

^a^ Cause of death may be multiple in some cases, hence %s may add up to >100

^b^ Data on patients who died in June 2003 missing

^c^ Data on patients who died in February and March 2007 missing; N = Total number of deaths in each year; n = number of deaths

## Discussion

The leading causes of mortality in children under the age of five years in this study are similar to findings from mortality studies from Nigeria, Kenya, Mozambique and South Africa [11–15]. It is also similar to studies on child deaths from Korle Bu Teaching Hospital (KBTH), Ghana in previous years [4,16]. What is different is the prominence of HIV, disappearance of measles as a major cause of death, the re-emergence of tuberculosis and reduction in typhoid fever deaths. The disappearance of measles as a major cause of disease attests to the effectiveness of the measles immunisation campaigns and efforts to meet the target for MDG 4 [17,18]. Neonatal deaths were not a prominent cause of the overall mortality unlike the national data and the study from KBTH which show a prominence of deaths from prematurity. This is mainly due to the service structure of the study hospital [4,16]. The Princess Marie Louise Children’s hospital is not attached to a maternity unit and it started a Special Care Babies Unit in 2009.

In this study, 127 deaths (9.7%) occurred in children aged five years and above. Nutritional disorders and Sickle cell disease were the main non-communicable diseases in adolescents. However, malaria, anaemia and HIV infection were the three most common diseases in the adolescent age group. This was also found in children aged 5–9 years but in addition septicaemia, malnutrition, tuberculosis and pneumonia were common and there were only three cases of typhoid fever. The study at KBTH in the 1980’s found that the major causes of mortality in the age group 5–12 years were typhoid fever, sickle cell disease and Burkitt’s lymphoma [4]. It is important to note that at the time, KBTH was a regional centre for treating Burkitt’s lymphoma. It is possible that currently, typhoid fever is being under-diagnosed, since the environmental conditions that foster the disease are still prevalent.

A study of post-mortems in adolescents in Accra found that typhoid disease, pneumonia and sickle cell disease were among the commonest causes of death among females. Abortions were an important cause of death too[19]. Our finding differs from this and it is probably because services for managing pregnancy-related complications and injuries at PML are limited. HIV was, however, a prominent cause of death in adolescents in this study. The WHO reports that while there has been a reduction in HIV infection worldwide, the condition is increasing in adolescents [20]. Therefore, while the world’s focus is currently on reducing mortality in under-fives, care must be taken not to neglect the health needs of older children and young person’s but rather hospitals like PML must be encouraged to expand their adolescent health services to deal with health problems in this age group. In addition, since the hospital runs a Sickle Cell Clinic, a more detailed audit of sickle cell deaths can help improve care and inform patient management at the clinic.

While malaria was the fifth major cause of deaths in the under-fives, it was the most common cause of death among children aged 5 years and above. Malaria in this age group needs to be closely monitored because it is expected that this age group will be most vulnerable if there are lapses in malaria control [21]. This is because the preventive measures currently in place, though effective, reduce exposure to the parasite in the early years which in turn reduces the immunity developed against the parasite. This becomes evident when the children are older. Thus data collection in hospital for surveillance purposes is essential.

The proportion of children dying from suspected septicaemia, pneumonia and meningitis rose during the period. The reason for this is unclear. It may well be that this is due to increased diagnosis as a result of a change in the labelling of unproven malaria or better recognition of the disease. The hospital also has the challenge of not being able to culture organisms on site and investigations for infection markers are largely unavailable. C-reactive protein is available from private laboratories but the cost is prohibitive for many patients. There have been studies to determine whether C-reactive protein and procalcitonin are effective biomarkers in the African region with mixed results particularly in malaria endemic regions [22,23].

The reliability of newer serological tests for the diagnosis of typhoid fever is more established but they are not available [24]. They may have improved the diagnosis of typhoid fever. Knowing the contribution of typhoid fever to mortality in this kind of setting is important because the disease can be prevented through vaccination programmes which have been established in some countries [25]. Therefore, strengthening laboratory services in the hospital will significantly improve the diagnosis of infections and determination of antimicrobial sensitivity. This is particularly relevant at this time when antimicrobial resistance and fake drugs are a matter of concern in Ghana.

It was also unclear whether the rise in these infections was due to the effect of undiagnosed HIV infection since the HIV status of all the children was not known. The 2013 HIV sentinel survey of pregnant women attending antenatal clinics at chosen sites within the country put the median HIV prevalence rate at 1.9% nation-wide and 2.7% in the Greater Accra Region [26]. However, the prevalence of HIV infection in hospital patients is often higher [27]. Testing all the children who will come to the hospital will be ideal as it will allow for early diagnosis of children with HIV infection. This will enable them to receive early treatment and prevent the disease from progressing. According to the hospitals protocol, all patients admitted to the ward with acute malnutrition should have HIV testing as well as a chest X-ray. This was started about 5 years ago. The study of children with Severe Acute Malnutrition (SAM) admitted to PML in 2010 found that 102 (75.6%) out of 135 children with SAM were tested for HIV and of those tested 21 (21%) had positive results for HIV infection [28]. The Ghana government is currently in the process of designing a programme to screen all children who attend health facilities for HIV infection.

Several population and hospital-based interventions ranging from immunisation programmes to improvements in the health services have contributed to the reduction in mortality from these diseases [29–31]. Increasing paediatric presence, development and use of case management protocols, training in the management of severe acute malnutrition and emergency triage may have also contributed to these changes [32]. However, additional effort should be put into preventing individual diseases and strengthening the health service to optimize care as other studies have shown [2,33–35].

We encountered some limitations during the study. We could not obtain sufficient information on patients who were discharged to determine case fatality of the diseases. We also encountered instances of missing data, which have been highlighted. Some patients had multiple diagnoses. However, since the cases were discussed at monthly mortality meetings around the time of death, a consensus was reached on several of the causes of death presented here. It is possible that we may have underestimated the number of pneumonias since the term Acute Respiratory Infection which is a broad term that refers to both upper and lower respiratory tract infections and includes pneumonia was among causes of death [36].

## Conclusions

There has been a decline in child mortality particularly from malaria, malnutrition and HIV in spite of a tripling of the admissions. However, the proportion of deaths from pneumonia in under-fives is rising. Deaths from diarrhoea, suspected septicaemia and meningitis have shown a fluctuating trend with only a modest decrease between 2012 and 2013 in spite of the introduction of pneumococcal and rotavirus vaccines. This stresses the need to strengthen the health system by providing better diagnostic services to aid the identification and proper management of infections. It is also important to understand the determinants of diseases such as pneumonia in the paediatric population in Ghana. Furthermore, there is a need to audit case management, improve data collection and management and conduct further studies to assess the contribution of undiagnosed HIV infection in mortality particularly in adolescents and school age children.
